# The first complete mitochondrial genome of Mitridae from *Mitra chinensis* (Neogastropoda: Volutoidea)

**DOI:** 10.1080/23802359.2020.1833770

**Published:** 2020-11-06

**Authors:** Ying Qiao, Shengping Zhong, Lianghua Huang, Yonghong Liu, Guoqiang Huang, Xiuli Chen

**Affiliations:** a Fourth institute of Oceanography, Ministry of Natural Resources, Beihai, China; b Institute of Marine Drugs, Guangxi University of Chinese Medicine, Nanning, China; c Guangxi Engineering Technology Research Center for Marine Aquaculture, Guangxi Institute of Oceanology Co., Ltd, Beihai, China; d Guangxi Key Laboratory of Aquatic Genetic Breeding and Healthy Aquaculture, Guangxi Academy of Fishery Sciences, Nanning, China

**Keywords:** Mitochondrial genome, *Mitra chinensis*, Volutoidea

## Abstract

Miters including *Mitra chinensis* are important fishery resources in China. However, the phylogenetic and taxonomic studies in Mitridae have been limited. In this study, we report the first complete mitochondrial genome of *M. chinensis*. The mitogenome has 16,238 base pairs (66.9% A + T content) and is made up of a total of 37 genes (13 protein-coding, 22 transfer RNAs, and two ribosomal RNAs), plus a putative control region. This study will provide useful molecular resources for addressing taxonomic and evolutionary issues in Neogastropoda.

The Neogastropoda Mitridae (miters) is a diverse gastropod family containing nearly 500 valid species around the world in 10 genera, which currently inhabiting mainly in tropical and subtropical shallow waters, particularly in Indo-Pacific ocean (Li and Li 2004). The taxonomy of this diverse group was traditionally inferred from shell morphology. Unfortunately, due to convergence and plasticity morphological characters, the taxonomy and classification of the Mitridae have been debated and a taxonomic revision has been reported recently (Li et al. 2005; Fedosov et al. 2018). Miters including *Mitra chinensis* are ecologically and economically importance species in Indo-Pacific benthic ecosystem (Briggs 1999). Complete mitochondrial genomes have been shown to be useful for phylogenetic inference and evolutionary studies at various taxonomic scales. Here, we report the first complete mitochondrial genome of Mitridae from *M. chinensis*, which will provide a useful genetic resource for phylogenetic and evolutionary studies in Neogastropoda.

Tissue samples of *M. chinensis* from six individuals were collected from GuangXi province, China (Beihai, 21.481455 N, 109.512299 E) using local trawl nets, together with the whole body specimen (#GR0353) were deposited at Marine biological Herbarium, Guangxi Institute of Oceanology, Beihai, China. The total genomic DNA was extracted from the muscle of the specimens using an SQ Tissue DNA Kit (OMEGA, Guangzhou, China) following the manufacturer’s protocol. DNA libraries (350 bp insert) were constructed with the TruSeq NanoTM kit (Illumina, San Diego, CA) and were sequenced (2 × 150 bp paired-end) using HiSeq platform at BGI Company, China. Mitogenome assembly was performed by MITObim (Hahn et al. 2013). The cytochrome oxidase subunit I (COI) gene of *Mitra cucumerina* (GenBank accession number: AY296839) was chosen as the initial reference sequence for MITObim assembly. Gene annotation was performed by MITOS (http://mitos2.bioinf.uni-leipzig.de).

The complete mitogenome of *M. chinensis* is 16,238 bp in length (GenBank accession number: MT415926) and contains conserved set of 13 protein-coding genes (PCGs), two ribosomal RNA genes, 22 transfer RNA genes, and a putative control region. The overall base composition of the mitogenome is estimated to be A 29.3%, T 37.6%, C 15.9% and G 17.1%, with a high A + T content of 66.9%, which is similar, but higher than *Bufonaria rana* (69.0%) (Zhong et al. 2020) which was closely related to Mitridae. The phylogenetic analysis inferred from the concatenated nucleotides sequences of 13 PCGs suggests that Mitridae has closely relationship with Bursidae and Volutidae (Figure 1). The complete mitochondrial genome sequence of *M. chinensis* was the first sequenced mitogenome in Mitridae, which will be useful for better understanding the phylogenetic and taxonomic classification of Volutoidea.

**Figure 1.**
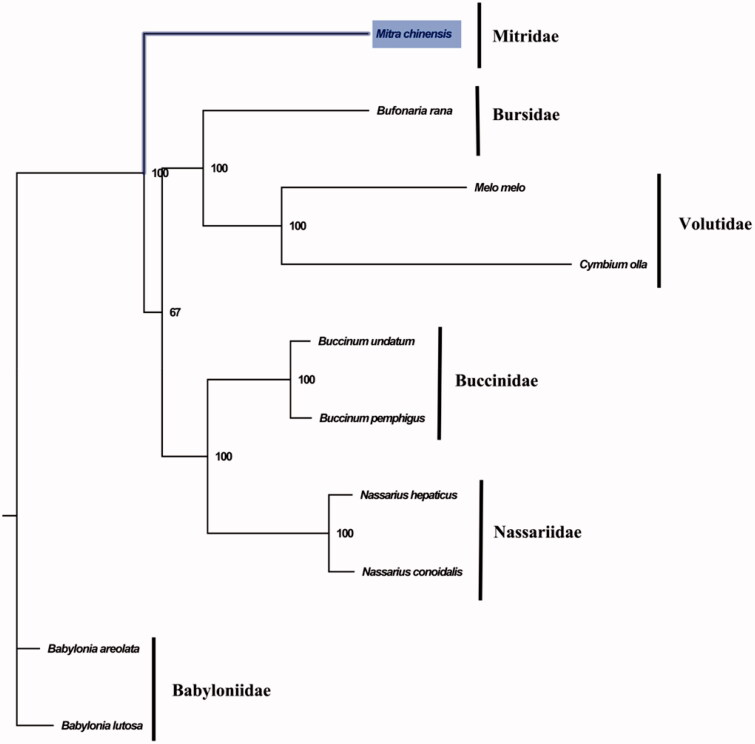
Phylogenetic tree of 10 species in Subclass Caenogastropoda. The complete mitogenomes were downloaded from GenBank and the phylogenic tree based on the concatenated nucleotide sequences of 13 mitochondrial PCGs was constructed by maximum-likelihood method with 100 bootstrap replicates. The bootstrap values were labeled at each branch nodes. The gene's accession number for tree construction is listed as follows: *Babylonia areolata* (NC_023080), *Babylonia lutosa* (NC_028628), *Melo melo* (MN462590), *Nassarius conoidalis* (NC_041310), *Nassarius hepaticus* (NC_038169), *Buccinum pemphigus* (NC_029373), *Buccinum undatum* (NC_040940), *Bufonaria rana* (MT408027), and *Cymbium olla* (NC_013245).

## Data Availability

The data that support the findings of this study are openly available in [National Center for Biotechnology Information] at [https://www.ncbi.nlm.nih.gov/nuccore], reference number [MT415926]. The raw data have been deposited into CNGB Sequence Archive (CNSA) of China National GeneBank DataBase (CNGBdb) with accession number CNP0001243.
